# Bladder Cancer and Probiotics: What Do We Know So Far?

**DOI:** 10.3390/cancers15235551

**Published:** 2023-11-23

**Authors:** Pedro Sánchez-Pellicer, Claudia Boix-Rodríguez, Adriana Hernández-Belmonte, Cristina de la Encarnación-Castellano, Alberto Mendiola-López, Eva Núñez-Delegido, Laura Navarro-Moratalla, Juan Agüera-Santos, Vicente Navarro-López, Juan Antonio Galán-Llopis

**Affiliations:** 1 MiBioPath Research Group, Faculty of Medicine, Catholic University of San Antonio de Murcia (UCAM), Campus de los Jerónimos 135, 30107 Murcia, Spain; pedro.sanchez@bioithas.com (P.S.-P.); ahbelmonte14@gmail.com (A.H.-B.); eva.nunez@bioithas.com (E.N.-D.); laura.navarro@bioithas.com (L.N.-M.); juan.aguera@bioithas.com (J.A.-S.); 2 Department of Internal Medicine, Hospital of Vega Baja, Carretera Orihuela–Almoradí s/n, 3314 San Bartolomé, Spain; claudiaboix@hotmail.com; 3 Infectious Disease Unit, University Hospital Vinalopó-Fisabio, Carrer Tonico Sansano Mora 14, 3293 Elche, Spain; 4 Department of Urology, General University Hospital Dr. Balmis, Investigación Sanitaria y Biomédica de Alicante (ISABIAL), Avenida Pintor Baeza 11, 3010 Alicante, Spain; cristina.delaencarnacion@gmail.com (C.d.l.E.-C.); albertomendiola.lopez@gmail.com (A.M.-L.); jagalanllopis@gmail.com (J.A.G.-L.)

**Keywords:** bladder cancer, microbiome, urinary microbiota, probiotics, *Lactobacillus*, *Bifidobacterium*, *Bacillus* Calmette–Guérin, *16S rRNA*, next generation sequencing

## Abstract

**Simple Summary:**

Recently, with the consolidation of the new high-throughput sequencing techniques, the old dogma that the bladder was sterile under normal conditions has been abandoned. Thus, the urinary microbiota in patients with bladder cancer could be involved in its pathophysiology, and its modulation with probiotics could be an interesting adjuvant therapeutic target. This narrative review aims to compile all the evidence to date provided by preclinical studies, animal assays, and clinical trials on the efficacy of probiotics that are either oral or injected into the bladder, thereby reducing the tumor size, preventing recurrences, modulating the immune system, etc.

**Abstract:**

Bladder cancer is around the 10th most diagnosed cancer, although has a considerable mortality. Recent research and new methodologies have discarded the historical dogma that the bladder (and urine) was sterile under normal conditions. Specifically, only a few studies have reported a detailed analysis of the urinary microbiota in patients with bladder cancer, thus exhibiting a remarkable variability due to the low biomass of the urinary microbiota and the influence of many factors. Nevertheless, this research shows us signals that urinary microbiota is a factor to be considered in the pathophysiology of bladder cancer. More importantly, probiotics could be useful as an adjuvant therapy to reduce the recurrence rate or increase the disease-free period after surgery. In vitro studies and animal assays have shown promising results, but the research in this context has also been scarce, and only a few studies have been conducted in humans. In summary, there is little evidence of the possible beneficial effect of probiotics in controlling the overgrowth of genera that could be involved in the carcinogenesis of bladder cancer. This narrative review aims to compile all the evidence to date on the therapeutic potential of probiotics injected directly into the bladder or orally administered.

## 1. Introduction

Bladder cancer (BC) is predominantly a uroepithelial carcinoma that is diagnosed in industrialized countries [1], thereby representing a spectrum of situations from recurrent chronic noninvasive tumors to advanced aggressive stages requiring multiple treatments [2]. Technically, uroepithelial carcinomas include tumors in the bladder, upper urinary tract (renal pelvis and ureters), and proximal urethra, but most of them are concentrated in the bladder. Histologically, most BC cases are pure uroepithelial carcinomas, although the diagnosis of histological variants is increasing [3,4]. Such a histological classification, together with the staging, presents important therapeutic and prognostic implications [5]. Therefore, the management of BC is dependent of the specific subtype. Tumors confined to the uroepithelium and lamina propria are considered as non-muscle-invasive BC (NMIBC) and are treated differently [6] from tumors that invade the muscle or beyond (stages T2, T3, or T4), which are referred to as muscle-invasive BC (MIBC) [7].

There were 573,278 new BC diagnoses (3% of all new cancer diagnoses) and 212,536 deaths caused by BC (2.1% of all deaths associated with cancer) worldwide in 2020 [8]. This means that BC became the 10th most diagnosed cancer. The incidence was higher in Europe and North America, although there was a high variability between countries, even within certain geographic regions [8]. Men are diagnosed four times more than women. Most cases are detected in patients over 60 years of age [9]. Tobacco smoking is the main risk factor, but constant exposure of the bladder to carcinogens and chronic bladder inflammation could explain the high incidence in certain regions [10]. In this regard, *Schistosoma haematobium* infection is a paradigm of causation of chronic inflammation. This infection leads to a process of inflammation that involves extensive tissue damage and granulomatous changes that predispose an individual to uroepithelial malignancy [11]. In addition, several single nucleotide polymorphisms (SNPs) have been found by genome-wide association studies (GWASs) to be genetic risk factors for BC, thus implying a hereditary component in this disease [12].

The World Health Organization (WHO) defines the concept of probiotics as “live microorganisms that, when administered in adequate amounts, confer a health benefit on the host”. This definition was ratified by the International Scientific Association for Probiotics and Prebiotics (ISAPP) in 2014 [13]. Probiotics are a safe treatment without significant adverse effects and have several beneficial properties that could be useful in the treatment or prevention of various diseases [14]. The efficacy of its immunomodulatory and antitumor properties has been studied in several types of cancer [15,16]. Considering the relevance of exploring new alternatives in the treatment or prevention of BC, this narrative review aims to provide current evidence regarding the efficacy of probiotics based on in vitro studies, animal assays, and human clinical trials.

## 2. Urinary Microbiota and Bladder Cancer

To understand how the modulation of the microbiome with probiotics could influence a health or diseases status, we must begin by understanding the characteristics—that is, the composition of that microbiome.

Regarding the bladder (urinary) microbiota, recent studies have discarded the historical dogma that both urine and the bladder were sterile under normal conditions [17]. New methodologies not based on microbiological cultures, such as the next generation sequencing (NGS) of the *16S rRNA* bacterial gene or metagenomic techniques, have provided robust evidence regarding the existence of a urinary commensal microbiota. Studies relating the urinary microbiota and pathological alterations to the bladder have been delayed more than those focused on other anatomical locations, because the bladder was not initially included in the Human Microbiome Project.

In contrast to gut microbiota, urinary microbiota contains a small number of bacteria, together with a significant presence of genetic material from the host due to shedding uroepithelial cells. Sample collection methods, sample volume, bacterial DNA extraction, *16S rRNA* gene primer selection, the taxonomic assignment of amplicons, and statistical analysis have been identified to imply a major influence on the interpretation of the results of a urinary microbiota analysis. This, which is already important in high biomass locations such as the gut, is even more significant in low biomass samples such as urine [18]. Therefore, these elements need to be controlled to reduce or minimize the probability of bias. What is more, this fact could explain the variability observed in the results reported to date regarding urinary microbiota in health and disease [19]. In this context, some consensus documents have been recently published to standardize the key aspects of urinary microbiota studies [20].

In most studies analyzing the bladder microbiota, the used sample has been urine from the middle portion after washing the genitals (clean-catch midstream urine). There are several considerations to correctly interpret the results using this sample [17]. The lower urinary tract is located near regions of high biomass such as the vagina, in the case of women, which could be an important source of microbiological contamination. This fact is less important in men; however, urine must pass through the urethra, and the bladder microbiota could mix with that from the urethra, with both not being identical. Even so, although it can not be a completely representative sample of the bladder microbiota, it is considered one of the most optimal samples due to its noninvasiveness and ease of collection [18].

Few studies have shown a detailed analysis of the urinary microbiota in patients with BC. Most of these studies have been conducted on clean-catch midstream urine using NGS of the *16S rRNA* gene and with a small sample size. Therefore, the nature and role of many relevant bladder bacteria in the initiation and progress of BC until now remains under investigation. However, the influence of urinary microbiota could be considered as a potential key factor in the onset and development of BC, as from the observation of the relationship between *Schistosoma haematobium* infection and squamous cell uroepithelial carcinoma, which could have its origin in tissue damage, chronic inflammation, and oxidative stress [11].

Let us begin to compile the evidence to date on the description of urinary microbiota in BC patients in terms of α and β diversity, as well as possible biomarkers.

Regarding α diversity, the results are very inconsistent when comparing the urinary microbiota of BC patients and healthy controls. In several studies, the α diversity indices of the urinary microbiota of BC patients and controls were not significantly different [21,22,23]. Moreover, no differences have been found between patients with NIMBC and MIBC [24]. On the other hand, there are studies reporting a reduction in the α diversity in BC patients [25,26]. Nevertheless, Zeng et al. highlighted the increased richness in urinary microbiota as a potential diagnostic biomarker of BC, thereby suggesting an overgrowth of procarcinogen bacteria, and a significantly greater α diversity in patients with NIMBC with a high recurrence compared to those with a low recurrence [23]. In fact, this was the first study to establish a relation between the urinary microbiota and clinical follow-ups in BC patients.

The dissimilarities at the β diversity level between the urinary microbiota of BC patients and healthy controls have been a more consistent finding [22,23,24,25]. This clearly suggests a urinary microbiota with a different structure and functionality in BC patients. Wu et al. reported a different urinary microbiota clustering in NIMBC patients with an elevated risk of recurrence and progression in comparison to those with an insignificant risk of recurrence and progression, but no differences were observed in relation to the tumor grade [22]. Meaningfully, Liu et al. found β diversity differences between tumoral and adjacent non-tumoral bladder tissue (with their study being the first microbiota BC tissue study) [27], and Pederzoli et al. also found differences between bladder tumoral tissue and urine-matched samples [28]. However, Hussein et al. did not find differences between NIMBC and MIBC patients [24].

The phylum Proteobacteria has been reported as the most abundant in urine [22,24,25,27] and tumoral [28] samples in BC patients in many studies. However, when searching for genera that specifically characterize the urinary microbiota of BC patients, there is a high degree of variability. Several genera such as *Fusobacterium* [21], *Acinetobacter* [22], *Actinomyces* [25], and *Bacteroides* [26] have been proposed as biomarkers of BC. In this regard, a recent systematic review of case-control studies published up to 2021 has concluded that there is no consensus regarding a BC biomarker bacterium according to sample type (urine or tumor tissue), and the role of uropathogens is inconsistent [29]. More studies on urinary microbiota in BC patients should be conducted, with better clinical design and appropriate sample sizes, according to current standardized protocols for sample collection and processing.

The main studies that have characterized the urinary microbiota of patients with BC are summarized in Table 1.

There are several reasons for considering the urinary microbiota as a crucial factor implicated in the pathophysiology of BC. Whether the changes in urinary microbiota that previous studies have demonstrated, either at the level of diversity or composition, are a cause or a consequence of the onset and development of BC is a question that should be further investigated. There is evidence that chronic inflammation is a key factor in many types of tumors, including BC, and the urinary microbiota is a key factor in the modulation or establishment of a bladder immune response [33]. Similarly, some beneficial bacteria could attenuate the inflammation established during the onset and development of BC. This enables the possibility of new strategies in the prevention and treatment of BC. Therefore, establishing the evidence regarding the therapeutic or preventive efficacy of probiotics in BC is an interesting issue. In this regard, a recent preclinical study has indicated a correlation between uroepithelial carcinoma BC and *Butyricicoccus pullicaecorum* [34]. This bacterium is an effective producer of butyrate (a short-chain fatty acid) with antitumor effects in bladder uroepithelial cells that are caused by influencing cell cycle, cell growth, apoptosis, and gene expression.

## 3. Bladder Cancer and Probiotics

### 3.1. Bladder Cancer and BCG-Based Immunotherapy

Although the *Bacillus* Calmette–Guérin (BCG)-based vaccine does not meet the exact WHO definition of a probiotic [13] and would not even fit with the postbiotic definition [35], its efficacy provides a reference for comparing it with some probiotic strains that have been assessed in patients with BC.

The BCG vaccine consists of an attenuated strain of *Mycobacterium bovis* that has lost its virulence in artificial cultures but retains its antigenic capacity. A beneficial effect on recurrences in BC patients was reported in 1976 for the first time [36], and from that moment, the intravesical injection of BCG is considered one of the most successful immunotherapies against the recurrence and progression of BC after transurethral resection [37]. Specifically, the NMIBC guideline from the European Association of Urology states that intravesical BCG injection after transurethral resection of the bladder tumor (TURBT) reduces the risk of tumor recurrence and is more effective than TURBT alone or TURBT and intravesical chemotherapy in patients with intermediate and high-risk tumors [6].

The exact mechanism of the BCG vaccine regarding his positive effect on the progression and recurrence of BC is partially unknown [38]. However, it has been evidenced that certain immune mechanisms against the tumor are activated. The urinary concentration of the tumor-necrosis-factor-related apoptosis-inducing ligand (TRAIL), a cytokine that induces apoptosis in malignant tumors without affecting healthy cells [39], was increased in patients responding to BCG treatment compared to non-responders [40]. Likewise, it has been observed that after the intravesical injection of BCG, there is a migration of neutrophils to the bladder that could cause a high secretion of the TRAIL [41]. Moreover, the BCG binds to the uroepithelium through a physicochemical interaction followed by damage to its mucopolysaccharide layer, thereby allowing the BCG to be remarkably close to the bladder wall, as it has been reported from animal assays [38]. Afterwards, the BCG is internalized more efficiently in bladder tumor cells [42], and then activation of the specific local and systemic immune system is produced. Uroepithelial cells and antigen-presenting cells (APC) are triggered, thus leading to the production of cytokines and causing the rearrangement of granulocytes and mononuclear cells in the bladder [43]. Neutrophils can mediate either the infiltration of CD4+ T cells or monocytes into the bladder or have a direct antitumor effect because they generate lytic enzymes and proapoptotic factors [40], as well as present phagocytic capacity. This could culminate in the development of a granuloma.

Nevertheless, intravesical BCG treatment presents several adverse effects [6]. *Mycobacterium bovis* infection can occur in less than 1% of cases; hence, caution must be exercised when BCG is administered in immunosuppressed patients [44]. Moreover, other less serious adverse effects include cystitis or cystitis symptoms, orchitis, arthralgia, arthritis, hematuria, prostatitis, general discomfort, and allergic reactions [6].

With existing evidence of the efficacy of the BCG vaccine, together with the knowledge of the partially elucidated mechanisms, we can broaden the horizon towards the search for probiotics with similar functionality but without the problems associated with adverse effects. In this way, the aim should be to obtain benefits that are comparable to BCG treatment but with a lower toxicity profile.

### 3.2. Bladder Cancer and Intravesical Probiotics: Preclinical Studies

**Figure 1:**
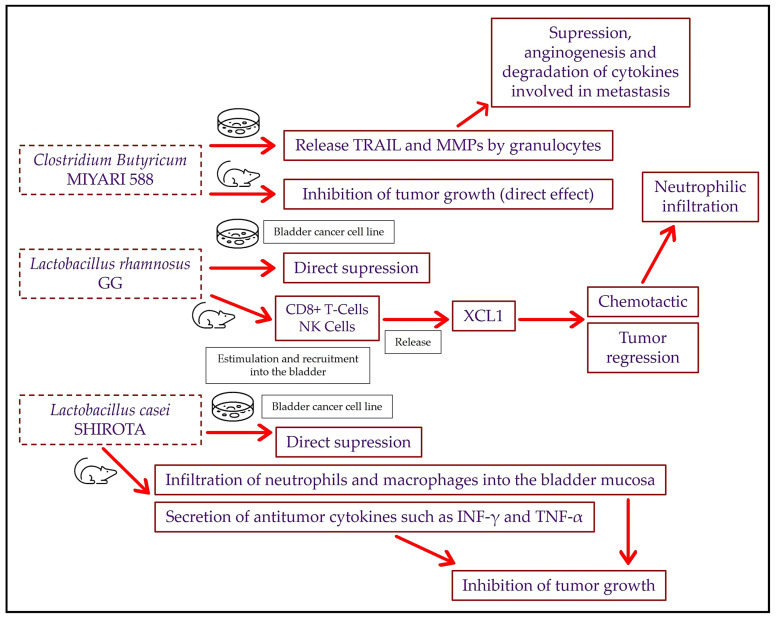
Mechanisms of action of intravesical probiotics described in murine models of bladder cancer and in vitro experiments. TRAIL: tumor-necrosis-factor-related apoptosis-inducing ligand; MMPs: matrix metalloproteinases; NK: natural killers; XCL1: chemokine ligand 1; INF-γ: interferon γ; TNF-α: tumor necrosis factor α.

Several preclinical studies have indicated that certain probiotic strains present beneficial anticancer effects specifically in BC by direct injection into the tumor site. By way of analogy, these probiotics are called “intravesical probiotics”. Shinnoh et al. experimented with the nontoxic strain *Clostridium butyricum* MIYARI 588 (CBM588) in different in vitro and in vivo studies [45]. The in vitro experiment revealed that CBM588 increased TRAIL secretion by granulocytes but without affecting its intracellular synthesis. The study also suggested that matrix metalloproteinase-8 (MMP-8) was one of the main molecules that influences TRAIL secretion by granulocytes. In fact, it has already been reported that MMPs have anticancer effects through the suppression of angiogenesis and the degradation of the cytokines involved in metastasis [46]. In addition, TRAIL release from granulocytes that was stimulated by this probiotic strain (as a supernatant after coincubation) caused the apoptosis of 253J-BV cells (a bladder cancer cell line), but it was also observed that CBM588 alone had little direct effect on the apoptosis of these cancer cells. However, a clear inhibition of tumor growth was observed when CBM588 was injected into the tumor using a model of BC in C3H/HeN mice with MBT-2 cells (a mouse transitional bladder carcinoma cell line) that were subcutaneously inoculated. The researchers highlighted that this bacterium (gut microbiota component), which has already been studied as a probiotic with different applications [47], could be a promising, safe, and effective treatment in BC patients (Figure 1).

Furthermore, Seow et al. published in 2002 a study of the efficacy compared to the BCG responses of some species of the potentially probiotic genus *Lactobacillus* in two BC cell lines [48]. In this regard, these researchers observed the effects of *Lactobacillus rhamnosus* GG and *Lactobacillus casei* Shirota on MGH and RT12 cell lines. MGH and RT12 cell line growth rates were significantly suppressed after exposure to both 10^7^ and 10^8^ colony-forming units (CFU)/L of *L. rhamnosus* GG between 48 and 72 h. Similarly, this outcome was observed with *L. casei* Shirota, but a cytotoxic effect was also evidenced in less than 24 h. At 72 h, preapoptotic cells were observed in both cell lines with 10^8^ CFU/L of *L. rhamnosus* GG. This was not observed with L. casei Shirota, because at that time there was an extensive cell death. Regarding the BCG vaccine, it also produced a reduction in cell growth when exposed to both cell lines but at a much lower percentage than both *Lactobacillus* species. Therefore, these *Lactobacillus* species were more effective than the BCG against the two BC cell lines. In relation to this, Takahashi et al. observed in an orthotopic animal assay of BC with MBT-2 cells (in this case, the implantation of tumor cells was directly into the bladder) that *L. casei* Shirota daily intravesical injection for 10 days was more effective than a treatment of equal duration with BCG [49]. This treatment with *L. casei* Shirota involved a greater reduction in the occurrence of BC and less tumor growth in these mice. Likewise, increases in the expression of antitumor cytokines such as the interferon γ (INF-γ) and the tumor necrosis factor α (TNF-α) were observed, as well as an infiltration of neutrophils and macrophages into the bladder mucosa that phagocytized the probiotic *L. casei* Shirota. Therefore, a clear increase in the local antitumor immune response was evidenced. The intravesical injection of some specific *Lactobacillus* strains could have an antitumor effect on BC through the effect observed by Takahashi et al., in addition to a direct cytotoxic effect. Importantly, the Takahashi et al. study [49] used a 10-day treatment scheme, whereas in healthcare practices administering a BCG intravesical injection, a 6-week schedule administered once a week is usually used. In this regard, in 2008, Seow et al. found out that the injection of BCG in healthy C57BL/6 mice for 6 weeks elicited greater cytokine production than the injection of *L. rhamnosus* GG, although the recruitment of NK cells into the bladder and draining lymph nodes was similar in both cases [50]. In 2010, Seow et al. once again used the same treatment schedule with both *L. rhamnosus* GG and BCG but in an orthotopic murine model of BC with the implantation of MB49 cells [51]. In this case, these researchers observed that *L. rhamnosus* GG probiotic therapy increased the levels of the chemokine ligand 1 (XCL1) compared to untreated mice. XCL1 is mainly produced by activated CD8+ T cells and NK cells, thus working as a chemotactic factor [52] and inducing tumor regression [53]. In addition, decreases in both the activated CD8+ T cells and NK cells in the spleen of mice treated with *L. rhamnosus* GG were reported. The authors then attributed the results to recruitment by the bladder. Furthermore, treatment with *L. rhamnosus* GG caused the mobilization of neutrophils into the bladder (as Takahashi et al. [49] observed with *L. casei* Shirota; see Figure 1).

Table 2 summarizes the main effects of these intravesical probiotic strains that have been evidenced in preclinical studies and murine assays.

### 3.3. Bladder Cancer and Oral Probiotics: Murine Assays and Human Clinical Trials

The antitumor effect of several oral probiotic strains in BC has also been evidenced in several studies in murine assays using ingestion. Asano et al. already showed in 1986 that mice consuming *Lactobacillus casei* (LC 9018 strain) reduced the size of a subcutaneously implanted BC tumor, and recurrences also decreased [54]. However, these researchers were unable to establish the mechanisms by which this *L. casei* strain inhibited tumor growth. In 2002, Lim et al. conducted a similar study, but with the probiotic *Lactobacillus rhamnosus* GG [55]. In this experiment, a group of C57BL/6J mice were inoculated with a MB49 bladder cancer cell line, and then a subgroup of mice were fed an oral suspension of *L. rhamnosus* GG. These mice showed a significant reduction in tumor size, especially when the initial tumor burden was small. Notably, this group of mice showed an increase in T cells that mostly infiltrated the tumor. The authors concluded that *L. rhamnosus* GG exerted its effected on the immune system, thus possibly crossing the intestinal barrier and reaching the lymphoid tissue where immune activation could occur. Another possibility may be that *L. rhamnosus* GG reached the tumor region and had a direct effect on the tumoral cells or that the strain induced the secretion of cytokines that could cause the infiltration of more immune cells into the tumor. Recently in 2023, Miyake et al. published a study with C3H mice that were subcutaneously inoculated with the BC cell line MBT2 and were treated with gemcitabine, cisplatin, and an oral probiotic mixture composed of *Lactobacillus casei* Shirota and *Bifidobacterium breve* strains [56]. The combination of the probiotic with gemcitabine and cisplatin increased the antitumor effect in terms of the reduction in implanted tumor size compared to these drugs being administered together or separately. Moreover, probiotic supplementation activated the processes of antigenic presentation involving the increased recruitment of cytotoxic T cells, which would explain the enhanced antitumor effect.

Regarding human clinical trials, Aso et al. reported in 1992 the efficacy of a preparation of approximately 10^10^ CFU/g of a *Lactobacillus casei* strain as a prophylactic to prevent recurrences in superficial BC [57]. These BC patients were randomized into a probiotic and a placebo group (25 and 23 subjects, respectively). The subjects in the probiotic group were treated with 1 g of the *L. casei* product 3 times a week for a year or until they presented a recurrence. Notably, the 50% recurrence-free interval in the probiotic group was 350 days, which was 1.8 times greater than that in the control group. In 1995, the same researchers carried out the same study, but with a multicenter design and with a larger sample size [58]. They included 61 BC patients in the probiotic group and 64 patients in the placebo group. The results confirmed the efficacy of the *L. casei* preparation for the prevention of recurrences in superficial BC after TURBT in both patients with multiple primary lesions and in patients with a single recurrent lesion. In the patients with a moderate risk of recurrence, the corrected cumulative recurrence-free rate one year after TURBT was over 25% significantly higher in the probiotic group than in the control group. Using the same probiotic preparation, Naito et al. published in 2008 a clinical trial with 102 superficial BC patients who received an intravesical epirubicin regimen after TURBT and with 100 superficial BC patients who received the same regimen of intravesical epirubicin after TURBT plus an oral *L. casei* preparation administered for one year [59]. The 3-year recurrence-free survival rate was significantly higher in the epirubicin plus *L. casei* group than in the epirubicin group without the administration of *L. casei* (74.6% versus 59.9%, respectively; *p* = 0.0234). The results of these clinical trials with *L. casei* provide evidence of the efficacy of this strain as an adjuvant treatment, thereby decreasing the risk of recurrence in patients with NMIBC. Nevertheless, this last study was commented on by Michael A. O’Donnell within the same journal [60], who criticized three aspects of the original paper. Firstly, he mentioned that 24 patients were withdrawn from the epirubicin plus *L. casei* group, and 7 were withdrawn from the epirubicin-only group, with no explanation of the causes. This would imply an imbalance in the study groups that may have affected the conclusions. Secondly, he criticized the nonblinded design of the study. Thirdly, he stated that the consumption of *L. casei* in Japan is especially important through fermented dairy products, and the study should be replicated in other countries with lower consumption.

An open-label, randomized, parallel-group clinical trial evaluating progression-free survival in BC patients treated with immunotherapy plus a probiotic mixture composed of several *Lactobacillus*, *Bifidobacterium*, and *Enterococcus* strains is currently running in order to evaluate the effect of these probiotics on the evolution of BC (it is registered in ClinicalTrials.gov; the identifier is NCT05220124).

Some other types of studies have provided evidence of the efficacy of probiotics in the prevention of BC. The influence of probiotics such as *Lactobacillus* strains on BC risk has particularly been studied in Asian countries, due to their great tradition of consuming fermented dairy products, which are rich in lactic acid bacteria (LAB). Ohashi et al., through a case-control study with 189 BC patients and 488 controls, observed that the odds ratios (ORs) obtained by a conditional logistic regression reported that smoking habits and the low consumption of Yakult and other Japanese fermented dairy products were more associated with BC status than healthy control status [61]. These authors concluded that the intake of fermented dairy products like Yakult prevent BC. It is noteworthy that, due to the Yakult fermentation process, the presence of viable LAB in its composition is assured, which is unlike in Western dairy products where the LAB would be lost due to more industrial processes. In this regard, a meta-analysis regarding the intake of fermented dairy products and the risk of diverse types of cancer was published in 2019 [62]. This meta-analysis included seven studies with 4673 BC patients and 408,049 healthy controls. An OR = 0.79 (95% CI = 0.68–0.92) was evidenced, therefore indicating a statistically significant protective effect.

Table 3 summarizes the main effects of oral probiotic strains that have been evidenced in murine assays and clinical trials.

## 4. Conclusions

This narrative review format has been used to summarize the current evidence on the efficacy of probiotics in BC in order to have more flexibility in the relevant topics developed, as well as to offer a paper outlining the state-of-the-art approaches. This is not only the main strength of the review, but also a limitation, as we have not addressed a more systematized perspective. However, considering the limited research to date on some of the topics, this would have been impossible. Due to the small number of studies performed to date, with only a few of them conducted in humans, we are still far from understanding the role of the microbiome in the origin of bladder cancer. There is also little evidence of the possible beneficial effect that probiotics could have as an adjuvant treatment acting on the microbiome by modulating it, as well as their role in controlling the overgrowth of those genera that could be involved in the carcinogenesis of bladder cancer. Studies using animal models have shown promising results, but there are few clinical trials that have been conducted in humans. However, the correlation between the consumption of foods rich in lactic acid bacteria and protection against BC is highly likely. We must continue investigating probiotic combinations in human beings that are effective in both preventing and reducing recurrences. In the future, clinical trials with strict designs and methodological quality should be considered, as there is still much to be explored in this field.

## Figures and Tables

**Table 1:** Main studies of microbiome in bladder cancer.

Study	Sample	Population	Key Results
Xu 2014 [30]	Urine	8 patients with uroepithelial carcinoma and 6 healthy controls	↑ *Streptococcus* in patients with uroepithelial carcinoma
Popovic 2018 [21]	Urine	12 BC patients and 11 healthy controls. All participants were men. BC Staging: 83% pTa and 17% pT1.	No differences in α diversity and richness. No differences in β diversity according to PCoA. Correlation of composition microbiota with age. ↑ *Fusobacterium* in BC patients. ↑ *Corynebacterium* in healthy controls.
Wu 2018 [22]	Urine	31 BC patients (26 NMIBC and 5 MIBC) and 18 healthy controls. All participants were men.	↑ richness in BC patients compared to controls. ↑ richness in NMIBC patients with high recurrence and progression compared to those with a minimal risk of recurrence and progression. No differences in α diversity. Differences in β diversity according to PCoA between BC and healthy controls and between NMIBC patients with high and low recurrence and progression. Proteobacteria most abundant phylum in BC patients. ↑ *Acinetobacter* in BC patients.
Bi 2019 [25]	Urine	29 BC patients (20 men and 9 women) and 26 healthy controls (15 men and 11 women). BC Staging: 34% pTa, 34% pT1, 18% pT2a, 7% pT2b, and 7% pT3a.	↓ α diversity in BC patients. ↑ CHAO1 in BC patients. Differences in β diversity according to PCoA between BC patients and healthy controls. ↑ Proteobacteria in BC patients. ↑ *Actinomyces europeans* in BC patients. ↑ *Lactobacillus* and *Bifidobacterium* in healthy controls.
Mai 2019 [31]	Urine	24 BC patients (18 men and 6 women). Comparison with 2 previous datasets.	Proteobacteria most abundant phylum in BC patients. Core urinary microbiota with 31 genera in BC patients. *Acinetobacter* characteristic genus of BC patients.
Liu 2019 [27]	Tumoral tissue	22 samples of tumoral tissue and 12 samples of adjacent nontumoral tissue. 5 NMIBC and 17 MIBC patients.	↓ Shannon index in tumoral tissue. Differences in β diversity according to PCoA between tumoral and no tumoral tissue. Proteobacteria was most abundant phylum in tumoral tissue. ↓ Firmicutes and Bacteroidetes in tumoral tissue. ↑ unclassified Brucellaceae; *Acinetobacter*, *Escherichia*/*Shigella*, *Sphingomonas*, *Pelomonas*, *Ralstonia*, *Anoxybacillus*, and *Geobacillus* in tumoral tissue. ↑ *Lactobacillus*, *Prevotella_9*, and Ruminococcaceae in nontumoral tissue.
Mansour 2020 [32]	Urine and tumoral tissue	10 BC patients (5 men and 5 women). 6 NMIBC and 4 MIBC patients.	↑ α diversity and richness in men with respect to women in tumoral tissue samples.
Pederzoli 2020 [28]	Urine and tumoral tissue	49 BC patients (36 men and 13 women) and 59 healthy controls (34 men and 25 women)	Differences in β diversity according to PCoA between tumoral tissue and urine. Firmicutes most abundant phylum in both tumoral tissue and urine samples. *Bacteroides*, *Akkermansia*, and *Klebsiella* most abundant genera in tumoral tissue. *Lactobacillus*, *Corynebacterium*, *Streptococcus*, and *Staphylococcus* most abundant genera in urine.
Zeng 2020 [23]	Urine	62 male BC patients (51 NMIBC and 11 MIBC) and 19 healthy controls. Follow-ups in 40 NMIBC patients.	↑ richness but not α diversity in BC patients. Differences in β diversity according to PCoA between BC patients and healthy controls. Differences in β diversity according to PERMANOVA test between NMIBC patients with high and low recurrence. ↓ α diversity correlated with longer recurrence-free survival. Total of 9 genera characteristically increased in patients with NMIBC and high recurrence.
Chipollini 2020 [26]	Urine	25 BC patients (17 MIBC and 12 NMIBC) and 10 healthy controls (globally 87% men).	↑ α diversity and richness in healthy controls. *Bacteroides* and *Faecalibacterium* characteristically increased in patients with invasive BC. *Bacteroides, Lachnoclostridium*, and Burkholderiaceae characteristically increased in patients with invasive BC.
Hussein 2021 [24]	Urine	43 BC patients (84% men and 67% NMIBC) and 10 healthy controls	No differences in α diversity between BC patients and healthy controls and between NMIBC and MIBC patients. Differences in β diversity according to PERMANOVA test between BC patients and healthy controls. Actinobacteria and Proteobacteria most abundant phylum in BC patients. *Actinomyces*, *Achromobacter*, *Brevibacterium*, and *Brucella* were significantly increased in BC patients. *Escherichia*/*Shigella*, *Faecalibacterium*, and *Lactobacillus* were significantly more abundant in healthy controls.

↑: Increase; ↓: Decrease; BC: bladder cancer; PCoA: principal coordinate analysis; NMIBC: non-muscle-invasive bladder cancer; MIBC: muscle-invasive bladder cancer.

**Table 2:** Main studies of bladder cancer and intravesical probiotics.

Study	Type	Probiotic	Key Results
Shinnoh 2013 [45]	Mice and in vitro	*Clostridium butyricum* MIYARI 588	Increase in TRAIL release by granulocytes. Reduction in tumor growth in a murine assay with a subcutaneously implanted BC cell line.
Seow 2002 [48]	In vitro	*Lactobacillus rhamnosus* GG and *Lactobacillus casei* Shirota	*L. rhamnosus* GG inhibited MGH and RT12 cell line growth between 48–72 h after treatment with 10^7^ and 10^8^ CFU/L. Cytotoxic effects were evidenced in less than 24 h with *L. casei* Shirota.
Takahashi 2001 [49]	Mice	*Lactobacillus casei* Shirota	BC orthotopic murine model of MBT-2 cells. *L. casei* Shirota daily intravesical injection for 10 days was more effective than treatment of equal duration with BCG, thus implicating less tumor growth. Increase in secretion of INF-γ and TNF-α, as well as infiltration of neutrophils and macrophages into the bladder mucosa that phagocytized *L. casei* Shirota.
Seow 2008 [50]	Mice	*Lactobacillus rhamnosus* GG	6-week scheme in healthy C57BL/6 mice. BCG intravesical injection caused greater cytokine production than *L. rhamnosus* GG. Recruitment of NK cells into the bladder and draining lymph nodes was similar using BCG and probiotic treatment.
Seow 2010 [51]	Mice	*Lactobacillus rhamnosus* GG	6-week scheme in an orthotopic murine model of MB49 BC cells. Increased XCL1 cytokine production and recruitment of neutrophils into the bladder that induced tumor regression.

TRAIL: tumor-necrosis-factor-related apoptosis-inducing ligand; BC: bladder cancer; BCG: *Bacillus* Calmette–Guérin; INF-γ: interferon γ; TNF-α: tumor necrosis factor α; NK: natural killers; XCL1: chemokine ligand 1.

**Table 3:** Main studies of bladder cancer and oral probiotics.

Study	Type	Probiotic	Key Results
Asano 1986 [54]	Mice	*Lactobacillus casei* (LC9018)	Reduced tumor size in a murine assay of subcutaneously implanted BC.
Lim 2002 [55]	Mice	*Lactobacillus rhamnosus* GG	Reduced tumor size in a murine assay of subcutaneously implanted BC, especially when the tumor was small. Tumoral infiltration of T cells.
Miyake 2023 [56]	Mice	*Lactobacillus casei* Shirota and *Bifidobacterium breve*	C3H mice were subcutaneously inoculated with BC cell line MBT2. Probiotic monotherapy had no significant effect in terms of reduction in the implanted tumor size, but antitumor effect was increased when it was combined with gemcitabine and cisplatin compared to these 2 drugs administered separately or in combination.
Aso 1992 [57]	Clinical trial	*Lactobacillus casei* 10^10^ CFU/g (1 g 3 times a week for1 year)	Efficacy as prophylactic to prevent recurrences in superficial BC. 25 subjects with probiotic and 23 with placebo. 87% men and 13% women. 90% papillary BC. 50% pTa and 46% pT1. 50% recurrence-free interval was 1.8 times greater in the probiotic group than in the placebo group.
Aso 1995 [58]	Clinical trial	*Lactobacillus casei* 10^10^ CFU/g (1 g 3 times a week for1 year)	Multicenter version of Aso 1992 study. 61 subjects with probiotic and 64 with placebo. 82% men and 18% women. 91% papillary BC. 61% pTa and 29% pT1. Corrected cumulative recurrence-free rate 1 year after TURBT was 25% higher in probiotic group than in placebo group.
Naito 2008 [59]	Clinical trial	*Lactobacillus casei* 10^10^ CFU/g (1 g 3 times a week for 1 year)	102 patients received an intravesical epirubicin regimen and 100 patients received the same regimen of intravesical epirubicin plus the oral probiotic preparation. 81% men and 19% women. 52% pTa and 48% pT1. The 3-year recurrence-free survival rate was significantly higher in the epirubicin plus *Lactobacillus casei* group than in the epirubicin-only group (74.6% versus 59.9%, respectively; *p* = 0.0234).

BC: bladder cancer; TURBT: transurethral resection of the bladder tumor.
